# A Novel Two-Step Method for Converting Lumbar Cortical Screw to Pedicle Screw: A Case Report

**DOI:** 10.7759/cureus.61454

**Published:** 2024-05-31

**Authors:** Logan Muzyka, Darsh S Shah, Michael T Koltz

**Affiliations:** 1 Department of Neurosurgery, Dell Medical School at The University of Texas at Austin, Austin, USA; 2 Department of Neurosurgery, University of Texas Medical Branch, Galveston, USA

**Keywords:** operative technique, new technologies in neurosurgery, new technology in spine surgery, spine surgery, case report, spine revision, pedicle screw, neuronavigation, cortical bone trajectory

## Abstract

The cortical bone trajectory (CBT) technique has emerged as a minimally invasive approach for lumbar fusion but may result in pseudoarthrosis and hardware failure. This report presents a case of successful pedicle screw revision in a patient with previous failed L2 and L3 fusion using a novel "two-step" technique, including (1) drilling a new trajectory with Medtronic EM800N Stealth MIDAS Navigated MR8 drill system (Medtronic, Dublin, Ireland) and (2) placement of Solera 4.75 ATS (awl-tapped screws) with navigated POWEREASE™ (Medtronic), described here for the first time. This method involves utilizing neuronavigation and specialized instruments to safely place pedicle screws through the path of the old cortical screw trajectory, addressing the challenges associated with CBT hardware failure.

## Introduction

Pedicle screw (PS) fixation is the traditional workhorse of thoracolumbar spinal instrumentation and a standard approach to the management of a variety of destabilizing pathologies of the spine. This technique has been described before in the literature [1]. However, despite its popularity, the technique may be associated with muscle and soft tissue injury and subsequently longer recovery times compared to newer minimally invasive spine (MIS) techniques [2].

One example of a new MIS technique is the cortical bone trajectory (CBT) screw technique. CBT, first introduced by Santoni et al. in 2009, has gained increasing popularity because it provides a less invasive option for lumbar fusion surgery [3]. In contrast to the PS technique, CBT has an insertion point at the junction of the superior articular process and pars, with a caudocephalad medial to the lateral path. This approach ensures engagement with at least three cortical areas: the dorsal cortex, the medial cortex of the pedicle, and the lateral wall of the vertebral body [4]. This technique allows for minimal soft tissue dissection with reduced risk of neurovascular injury [5].

As CBT continues to grow in popularity among spine surgeons, there may be an increased necessity to salvage a loose or compromised cortical screw for several reasons, including errors in screw placement, hardware failure, or screw loosening [6]. Another major limitation of CBT is that there is not adequate space within the pedicle if the screw comes loose, as compared to a traditional PS, which allows more room for upsizing. Thus, spine surgeons must be aware of techniques for screw salvage, especially considering the anatomical challenges. A few studies have shown PS revision of CBT to have high efficacy [6,7]. However, no report to our knowledge has outlined in detail the surgical technique for CBT revision with PS using neuronavigation. If it is possible to drill across the existing trajectory and then insert the PS, it would eliminate the need for tapping a new hole or redoing insertion over a wire. Thus, in this paper, we report a case of a patient with prior history of multiple back surgeries, including an L2 and L3 fusion with cortical screw placement, who received a PS revision of the fusion.

## Case presentation

A 79-year-old man presented to the clinic with intractable back and R > L leg pain after multiple prior back surgeries, including an L2-L3 lumbar fusion with cortical screws. The patient’s pain had progressed to requiring a walker for ambulation and was unresponsive to activity modification, pain management, and physical therapy.

The patient’s physical exam revealed pain with extension and flexion, decreased range of motion, 3/5 strength, and 2/4 reflexes in the right lower extremity.

**Figure 1:**
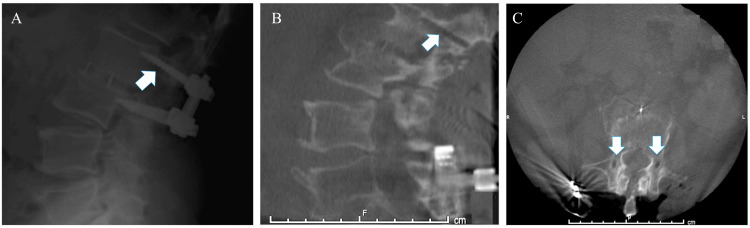
Preoperative standing upright lateral X-ray shows cortical screws with hypodensity surrounding the screws superiorly concerning for pseudoarthrosis (A). A preoperative lumbar CT scan shows the caudal-to-cranial (B) and medial-to-lateral (C) path of removed cortical screws in a sagittal and axial frame.

Radiographs at initial presentation revealed pseudoarthrosis at the CBT level L2-L3 (Figure 1) and severe adjacent segment disease at L3-L4 with right L3-L4 subacute disc herniation. The patient consented to undergo revision of his L2-L3 CBT construct to PS with extension down to L4 given the decompression needed to treat the adjacent segment’s pathology.

Technique

**Figure 2:**
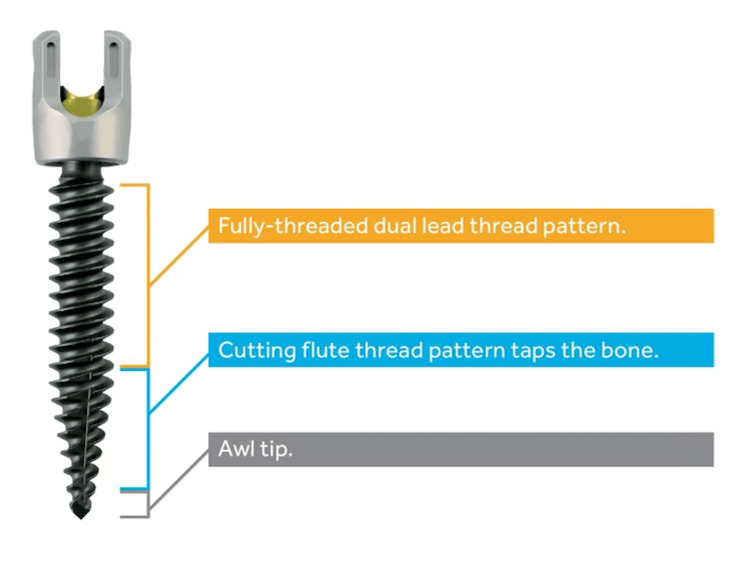
Medtronic Solera 4.75 ATS (awl-tapped screw). Authorization for the use of this image was secured through the marketing representatives of Medtronic Brain and Spine Therapies.

**Figure 3:**
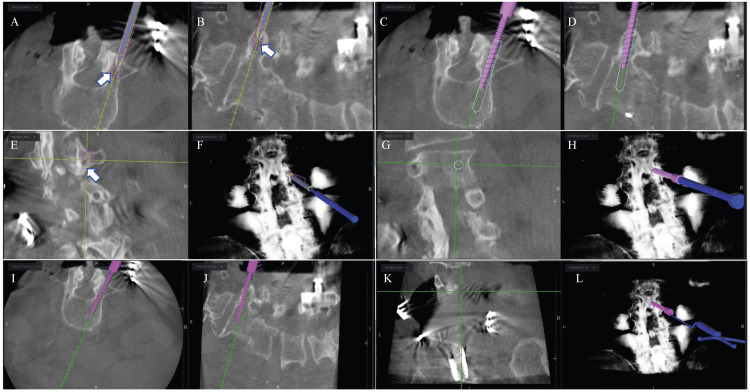
Intraoperative neuronavigation showing real-time virtual screw placement converting removed cortical screws to pedicle screws. Top row: Planning the entry point of the traditional pedicle screw. The white arrow points to the location of the previous cortical screw (hypodense). Axial representation (A) for the entry point of the virtual screw starts lateral to the entry of the cortical screw. Sagittal representation (B) shows the trajectory of the pedicle screw to be parallel to the superior endplate. Coronal representation (C) for the entry point of the virtual screw starts superior to the entry of the prior cortical screw. X-ray representation of the pedicle screw trajectory is shown in image D. Middle row: Screw advancement through the pedicle in the axial frame (E), parallel to the superior endplate in sagittal frame (F), in the coronal frame (G), and an X-ray representation (H). Bottom row: Final position of the pedicle screw in the axial frame (I), sagittal frame (J), coronal frame (K), and X-ray representation (L).

**Figure 4:**
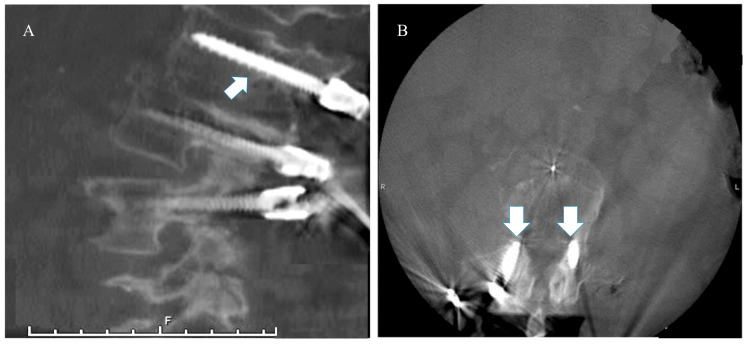
The intraoperative CT scan shows the conversion of the cortical screw to the pedicle screw (white arrow) in the sagittal (A) and axial (B) frame. The new pedicle screw follows a medial-to-lateral path and is parallel to the superior endplate.

The patient underwent surgery with neuronavigation and neuromonitoring. Neuronavigation included the Stealth-Midas electric system (Medtronic, Dublin, Ireland) with 3 mm Legend Match Head-Fluted for pedicle axis and POWEREASE™ with Solera 4.75 ATS (awl-tapped screws) (Medtronic) pedicle screw for screw placement. Neuromonitoring allows current to be applied through the POWEREASE™ drill for real-time stimulation during hardware placement. The patient's previous midline incision was opened and old hardware was exposed. The exposure was carried out laterally to expose the traditional landmarks for standard pedicle screw insertion at L2, L3, and L4. The patient’s previous hardware was removed in total and intraoperative neuronavigation spin was performed uneventfully (Figure 1). Using O-arm neuronavigation (Medtronic), traditional pedicle screws were placed into L2, L3, and L4 vertebral bodies crossing the trajectories of the previously placed cortical screws (Figure 2). The entry point and access into the distal pedicle were done with the Medtronic electric drill with a 3 mm matchstick drill bit under neuronavigation. The screen projection displayed the final screw size to assure no breaches, as opposed to the 3 mm drill bit. This technique allows for the patient’s previously placed cortical screw tract to be crossed without falling into the old path when the final screw was placed. The final screw was placed next using POWEREASE™ and new Solera 4.75 ATS (Medtronic, Figure 2) with continuous neuromonitoring stimulation. This technique allows for placing significantly larger screws despite previous cortical screw tracks: screws at L2 were 6.5 x 55 bilaterally; L3, 6.5 x 60 bilaterally; and L4, 6.5 x 60 on the right and 6.5 x 55 on the left. Finally, a repeat intraoperative O-arm neuronavigation spin confirmed adequate placement of the new pedicle screws (Figures 3, 4).

**Figure 5:**
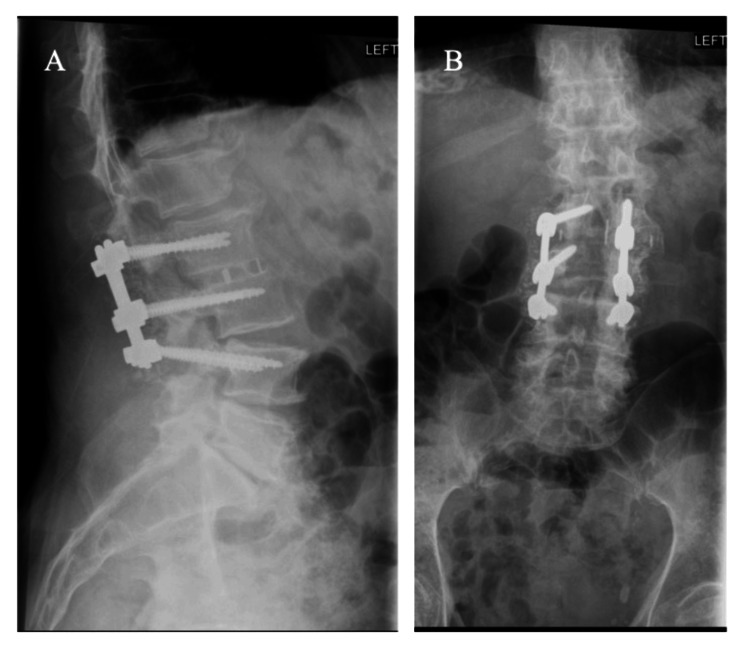
Three months postoperative standing lateral (A) and anteroposterior (C) X-ray shows successful conversion of the cortical screw to the pedicle screw construct without evidence of pseudoarthrosis.

Three months postoperatively, the patient reports significant improvement in his back pain and is now able to ambulate without a cane. X-ray imaging shows good construct formation without radiolucency to suggest pseudoarthrosis or screw loosening (Figure 5).

## Discussion

**Figure 6:**
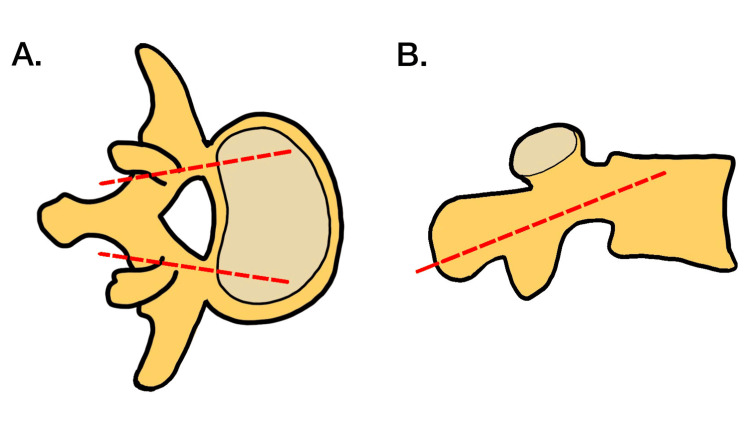
Demonstration of cortical bone trajectory with (A) a lateral path in the transverse plane and (B) a caudocephalad path in the sagittal plane. Image adapted from Laratta JL, Shillingford JN, Pugely AJ, et al. Accuracy of cortical bone trajectory screw placement in midline lumbar fusion (MIDLF) with intraoperative cone beam navigation. J Spine Surg. 2019;5(4):443-50. doi: 10.21037/jss.2019.09.10.

Santoni et al. first proposed the CBT technique as an alternative to the traditional PS fixation technique to maximize screw purchase [3]. The laterally directed trajectory in a caudocephalad path engages only cortical bone in the pedicle without the involvement of the vertebral body trabecular space. Additionally, CBT utilizes a shorter and slimmer screw design, which has a specific trajectory that maximizes contact with the cortical bone (Figure 6) [3,8]. Several studies have validated the excellent outcomes of this technique in the literature. These studies have shown that the more medial CBT limits dissection lateral to the facet joints as is the case in PS fixation, resulting in reduced operative time, blood loss, and short-term morbidity in patients undergoing lumbar fusion with this technique [9-13]. Biomechanical differences between CBT and PS fixation have largely been inconclusive. Some studies point to improved resistance to toggling and pullout forces in CBT, while others report biomechanical equivalency [6,14]. Notably, a meta-analysis by Hu et al. indicated that CBT screws tend to experience higher stress levels compared to PS screws, potentially increasing the risk of implant failure [15]. CBT screws, with their focused cortical bone contact, may lead to localized stress concentrations, potentially increasing the risk of screw loosening or pullout. In contrast, PS screws distribute loads evenly across the pedicle, vertebral body, and adjacent structures, reducing stress concentrations and the risk of implant failure. PS screws engage both cortical and trabecular bone, providing comprehensive fixation and enhancing stability, which may reduce the likelihood of screw failure.

While studies evaluating CBT have been promising, justifying the increasing use of the technique, long-term studies of this approach have shown a relatively high failure rate [16-19]. Akpolat et al. conducted a cadaveric study in 12 vertebrae from six cadaveric spines and found that CBT screws had a significantly decreased fatigue performance when compared to the PS screw control [12]. Similarly, Liu et al. found that the slim CBT screws were highly stressed compared to their PS counterparts, indicating that they had a higher propensity for failure due to loosening or cracking [20]. Thus, given the chance of failure associated with CBT screws, it is important for a spine surgeon to have techniques for revision in their repertoire.

PS rescue of failed CBT screws has been found to have excellent efficacy. Calvert et al. found that pedicle rescue screws retained an average of 65% of the original cortical screw pullout strength. Moreover, they found no significant differences in stiffness testing in flexion/extension, lateral bending, and axial rotation between the original CBT screws and the subsequent rescue PS [6]. Similarly, Zhang et al. found that the PS screws retained adequate insertional torque, pullout strength, and fatigue performance when used to revise CBT screws in osteoporotic spines [7]. Thus, PS revision of failed CBT screws provides a viable option for spine surgeons and should be employed when needed. Moreover, the importance of initial screw selection is emphasized, especially in scenarios prioritizing biomechanical stability and load distribution, where PS screws may be preferred for their ability to evenly distribute loads and reduce the risk of implant failure. Furthermore, the importance of initial screw selection is emphasized, recognizing that biomechanical stability and load distribution are consistently fundamental priorities. For this reason, pedicle screws are often favored for their capacity to evenly distribute loads and mitigate the risk of implant failure.

In the case presented above, we show the technique for the revision of a failed CBT screw construct to a traditional PS construct utilizing neuronavigation. The authors feel that this technique allows for the traditional PS construct to be made in a safe manner despite a previous cortical screw tract within the targeted pedicles by combining the “standard of care” intraoperative anatomic landmarks with neuronavigation software (Stealth, Medtronic) and hardware (Stealth-Midas electric system with 3 mm Legend Match Head-Fluted for pedicle axis and POWEREASE™ with Solera 4.75 ATS) technology. Neuronavigation allows for the use of the navigated drill bit to cross the path of the old cortical screw trajectory through the pedicle with the new traditional PS trajectory. The navigated POWEREASE™ with ATS allows the placement of the final PS in one step using continuous neuromonitoring and continuous visual feedback from the neuromonitoring screen. Finally, an intraoperative CT scan post hardware placement confirms satisfactory hardware placement prior to leaving the operating room, confirming the successful completion of the planned procedure.

## Conclusions

Transitioning from CBT to PS represents a crucial skill for spine surgeons, especially given the widespread adoption and benefits of minimally invasive techniques. When hardware failure occurs with less invasive methods like CBT, it is essential for surgeons to have safe salvage options. Our case shows it is safe and effective to drill across an old trajectory and directly follow with ATS, eliminating the need to tap a new hole or do over a wire. Further studies implementing the two-step method are necessary to support the standard use of this technique. This case underscores the importance of addressing complications associated with CBT screws and emphasizes the need for surgeons to proficiently revise failed CBT constructs with traditional PS screws.
